# Conducting Polymeric Nanocomposites with a Three-Dimensional Co-flow Microfluidics Platform

**DOI:** 10.3390/mi10060383

**Published:** 2019-06-07

**Authors:** Xiaodong Ma, Yuezhou Zhang, Korbinian Weisensee

**Affiliations:** 1 Xi’an Institute of Flexible Electronics & Xi’an Institute of Biomedical Materials and Engineering, Northwestern Polytechnical University (NPU), Xi’an 710072, China; 13851280968@163.com; 2 Department of Pharmaceutical Science Laboratory, Åbo Akademi University, 20520 Turku, Finland; zyuezhou@126.com

**Keywords:** polymeric NPs, microfluidics, Ac-DEX, Sp-Ac-DEX, PLGA, chitosan, polymer concentration, flow rate, inner capillary opening

## Abstract

The nanoprecipitation of polymers is of great interest in biological and medicinal applications. Many approaches are available, but few generalized methods can fabricate structurally different biocompatible polymers into nanosized particles with a narrow distribution in a high-throughput manner. We simply integrate a glass slide, capillary, and metal needle into a simple microfluidics device. Herein, a detailed protocol is provided for using the glass capillary and slides to fabricate the microfluidics devices used in this work. To demonstrate the generality of our nanoprecipitation approach and platform, four (semi)natural polymers—acetalated dextran (Ac-DEX), spermine acetalated dextran (Sp-Ac-DEX), poly(lactic-co-glycolic acid) (PLGA), and chitosan—were tested and benchmarked by the polymeric particle size and polydispersity. More importantly, the principal objective was to explore the influence of some key parameters on nanoparticle size due to its importance for a variety of applications. The polymer concentration, the solvent/non-solvent volume rate/ratio, and opening of the inner capillary were varied so as to obtain polymeric nanoparticles (NPs). Dynamic light scattering (DLS), transmission electron microscopy (TEM), and optical microscopy are the main techniques used to evaluate the nanoprecipitation output. It turns out that the concentration of polymer most strongly determines the particle size and distribution, followed by the solvent/non-solvent volume rate/ratio, whereas the opening of the inner capillary shows a minor effect. The obtained NPs were smooth spheres with adjustable particle diameters and polymer-dependent surface potentials, both negative and positive.

## 1. Introduction

Fessi and co-workers first developed the nanoprecipitation technique—or as they termed it, the solvent displacement approach—for nanoparticle fabrication [1]. Nanoprecipitation displays multiple advantages, including being rapid and easy to execute. In general, anti-solvent nanoprecipitation formation is prompt and requires two miscible solvents. One is a good solvent for the solute in use, while the other is a poor solvent which hardly dissolves the solute. Among a variety of precipitatable materials, polymers have gained the highest popularity, mainly due to their potential applications.

Biodegradable polymer nanoparticles (NPs) are of particular interest since they can deliver a combination of various therapeutic ingredients [2], integrate targeting moieties [3], protect active ingredients from degradation [4], control the release of ingredients, adsorb diesel oil contaminants from organic solutions [5], stabilize iron oxide NPs through electrostatic or steric forces by the natural polymer [6], and form multi-layered polymeric honeycomb spheres to hold Fe_3_O_4_ NPs [7]**.** The prevailing involvement of polymeric particles/spheres in a diversity of applications highlights the necessity of particle fabrication. After the mixture of a polymer in a good solvent and a poor solvent is formed, the precipitation is believed to take place in three stages: nucleation, growth by aggregation, and growth by coagulation that leads to the formation of a colloidal suspension of polymer particles in the anti-solvent almost instantaneously [8]. Important goals in NP production are ensuring homogeneous particle composition and minimizing the particle size distribution. Of these, the particle size distribution likely has the most significant effects on potential medical applications since NPs’ size determines their migration and transformation through tissue, and particles differing by size are internalized by cells using different mechanisms [9]. The release of the active pharmaceutical ingredient (API) from NPs, for instance, occurs by either simple diffusion or nanoparticle degradation, and this is heavily dependent on the particle size [10]. Smaller nanocomposites represent a greater surface-area-to-volume ratio; thus, they are to release the drug much more rapidly, which may result in high local API concentrations that could potentially lead to unwanted side effects. Accordingly, a broad size distribution means poor control over the API delivery and release, making it harder to determine a suitable therapeutic dose for the patient [11]. Such failure of control is driving the demand for production methods that reduce polydispersity.

Nanoparticulation can be implemented through many platforms/techniques [12]. The standard protocol is batch processes [13], which usually has several benefits: very efficient fabrication of a large volume of nanomaterial and conceptually easy in that injection can be performed by one-pot pouring of the organic phase into the aqueous solution. However, this straightforwardness is heavily compromised by a key drawback: it is hard to scale up a batch process with perfectly homogeneous mixing, leading to large batch-to-batch variation [14]. Some trivial issues, such as the scale of the magnetic stirrer and the point of dropwise addition of the organic phase, can dramatically affect both the particle dispersity and the size.

There is no doubt that microfluidics represents an indispensable alternative for nanoparticle production [15] given its advantages in addressing the upscaling difficulties, batch-to-batch variation, and control of the nanomaterials’ physicochemical properties [16] over the conventional “bottom-up” or “top-down” approaches. These improvements associated with microfluidics methods are due to the well-established synthesis conditions and sufficient control of the mixing process, thereby producing homogeneous nanomaterials with the desired merits. Microfluidics miniaturizes capillary networks and enables exquisite control over the flows of multiple fluids in micrometer-sized channels; therefore, it is capable of rapid and uniform mass transfer and consequently produces nanomaterials in a high-throughput manner when proper materials and fabrication methods are applied [17]. The size of the particles precipitated can strongly depend on the aqueous-to-organic ratio, which is precisely tunable through an injection pump in a microfluidics process. Furthermore, the production can be scaled up by running several microfluidics chips in parallel. The production of NPs using a microfluidics platform has been monitored with the help of synchrotron small-angle X-ray scattering (SAXS) [18]. Combined with the continuous in situ synthesis of nanomaterials, a microfluidics platform therefore favors industrial upscaling.

To date, microfluidics devices have been diversified for the preparation of a variety of micro-/nano-sized materials, including hydrogels [19]; inorganic, polymeric micro- and nanoparticles, as well as hybrid particles; cell encapsulation [20]; even tissue engineering [21]. Putting the above-mentioned advantages together, microfluidics devices provide an ideal platform for the high-throughput synthesis of micro/nanomaterials. Polydimethylsiloxane (PDMS) or silicone is widely used in most microfluidics devices for NP synthesis [22] because it is easy to work with, economical, and transparent [23]. Nevertheless, as a silicon-based organic polymer, the compatibility of PDMS with organic solvents is of great concern considering the potential of PDMS-based microfluidics devices in a variety of applications, particularly when the solutes in use are only dissolvable in organic solvents. Three aspects determine the compatibility of PDMS with a solvent, including PDMS swelling in a solvent, the absorption of solutes into PDMS, and the dissolution of PDMS oligomers in a solvent. Among these, it has been suggested that the swelling of PDMS has the greatest influence [24]. This is compounded by NPs made from polymers, such as hypromellose acetate succinate [25], which tend to precipitate on the hydrophobic channel walls of PDMS, leading to channel blocking. These challenges can be met through the surface modification of the PDMS channels. However, the coating tends to become detached over time and the antifouling no longer functions. In addition, PDMS itself is viscoelastic [26], suggesting that high pressure applied to PDMS-based microfluidics devices may result in local deformation invisible to the naked eye and introduce unstable flow in the microfluidics device channel, which is fatal for obtaining monodisperse NPs. Further, typical PDMS-based microfluidics devices suffer from low productivity, <7.2 g∙day^−1^ of NPs, for instance, putting them far behind the productivity required in the pharmaceutical industry. Efforts such as a sophisticated microfluidics approach to maintain the three-dimensional (3D) focusing flow patterns [27] have been attempted to prevent localized polymer aggregation near the PDMS microfluidics channel walls, and devices based on glass materials are desired in order to be compatible with the organic solvent [28].

In this article, we firstly elucidate the assembly process of the microfluidics chip made of glass which we have used in our experiments since 2015 [16]. Then, a simple workflow connected with a microscope is detailed. To demonstrate the reliability and robustness of this platform, four commonly used biomaterials—acetalated dextran (Ac-DEX), spermine acetalated dextran (Sp-Ac-DEX), poly(lactic-co-glycolic acid) (PLGA), and chitosan—were fabricated into NPs using the as-prepared microfluidics chip and auxiliary apparatus. The effect of polymers, the inner-to-outer flow rate/ratio, and the opening of the tapered inner capillary on the size and distribution of the obtained particles was investigated.

## 2. Materials and Methods

### 2.1. Building a Chip for Microfluidics

#### 2.1.1. Syringe tip fabrication

All materials and accessories used for microfluidics chip assembly are listed in Table 1. Take two syringe tips (= 1)) and remove all the plastic parts with a knife or soften them through heating so that only the metal part remains (= 1a)). These will be used as the inlet and the outlet of the microfluidics system. Cut two opposing V-shaped holes on the bottom of the plastic part of the remaining one. The holes should be of different size. The first one covers the size of the inner capillary, and the second one covers the size of the outer capillary; this is so that the tip can stand on top of the capillaries with the inner one on one side and the outer one on the other side (= 1b)). Make the holes as big as necessary but as small as possible (Tip 1).

Tip 1: Use (2) and (3) to check the size by placing the syringe on top of them and seeing if it can stand alone while touching the ground. If the holes are too big, glue can flow inside and block the system.

#### 2.1.2. Inner capillary tapering

In order to form a tip with the inner capillary, it is placed with its middle in the puller (so that the glass tube is heated in the middle). Maintain the configured settings for a proper tip shape. When tightening the screws to keep the glass tube in place, make sure that the moving part of the puller is as close to the right-hand side as possible. Tighten the screws firmly but do not break the glass tube. After the glass tube is pulled apart, “sharpen” the tip on the sandpaper until a proper tip size of around 100 nm is achieved (= 2a)) (this equals to 1 cm on the screen of the microscope in the microfluidics lab) (Tip 2). Make sure that the nozzle shows an even line (Tip 3). Use pressured air to get rid of glass particles on the outside of the formed tip. Prepare the other half of the inner capillary likewise for either further usage or as a backup in case there are issues with the first one.

Tip 2: Keep the sandpaper in the other hand instead of placing it on the table in order to reduce the pressure applied to the glass tip.

Tip 3: Place the glass tube between the thumb and forefinger and roll it between them while moving over the sandpaper so the tip has an even form.

#### 2.1.3. Outer capillary Truncation

Cut the outer capillary with the glass cutter in the middle. Keep one for further usage. Cut a small piece from the other one, which will be used further as a bridge between the inlet and the inner capillary of the microfluidics system (= 3b)) and keep the longer part (= 3a)).

#### 2.1.4. Put All Parts Together

**Figure 1:**
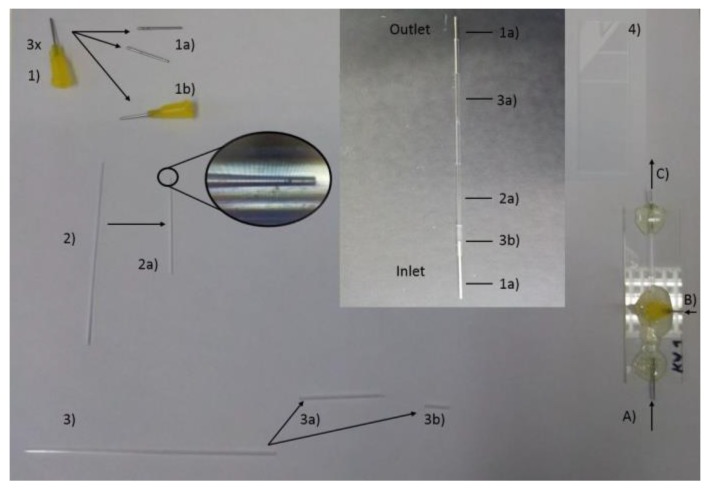
The building blocks for the microfluidics chip. (**1**) The inlet and outlet needle of flow; (**2**) Tapered inner capillary; (**3**) Outer capillary; (**4**) Assembly of individual parts on a glass slide.

Align all created pieces together as shown in the middle of Figure 1 (Tip 4). Place all the parts on the glass slide (4) and readjust all pieces that slipped out of position. Put (1b) now on top of the part where the outer capillary (3a) starts to cover the inner capillary (2a) (with the holes to its contributing partner). Prepare the glue by mixing the two components with each other. When the glue starts to get less viscous, it is ready to use (Tip 5). Use the stick which was used for mixing the glue and collect some glue on its tip. Let the glue drop naturally from the top of the stick to the syringe tip (1b) so that it can flow to the work surface (Tip 6). Do this until the whole bottom part of the syringe tip is covered (Tip 7). Continue now by adding glue to the inlet and the outlet part (Tip 7). Try to avoid moving the system now and let the glue solidify overnight. In the end, a co-flow 3D microfluidics chip is made, as displayed in Figure 1 (Bottom right).

Tip 4: Make sure that (1a) and (2a) touch each other in order to reduce the hollow areas in the system.

Tip 5: Use a piece of paper/cartridge to mix the glue. Wear gloves because it is sticky. When the glue viscosity decreases, one can also feel a little bit of warmth on the other side of the paper/cartridge. If the glue reaches this point, be quick, but there is no need for excessive haste.

Tip 6: If the glue is not viscous enough it will run underneath the syringe tip and block the system; it should have properties similar to liquid honey or a small exothermic reaction should be observed, implying that a crosslink reaction has occurred between epoxy resin A and resin B.

Tip 7: Use as much glue as needed, but as little as possible.

#### 2.1.5. Check the Chip

Before using the newly created chip, check the following things:

- Did the tip of the inner capillary remain undamaged? View the tip under a microscope in the microfluidics lab.
  - It is damaged → start anew.
- Does the system have a leakage/blockage? Run the system with ethanol to (A) and pure water to (B) with a flow rate of 2:40 mL/h (inner/outer), and collect the NPs from (C).
  - Blockage → start anew.
  - Leakage → dry the chip and apply some glue to the leaking areas; let the glue solidify and try again.

During the experiments, persist with one chip in order to minimize potential influences on different outcomes of the same experiment until it is unrepairable.

### 2.2. Materials for Nanofabrication

Four polymeric materials—Ac-DEX, Sp-Ac-DEX, PLGA, and chitosan—were used to validate the availability and generality of our in-house-built microfluidics platform for nanofabrication.

Ac-DEX was synthesized from dextran according to a published procedure with minor modifications [29]. In brief, an oven-dried flask was charged with dextran (M_W_ = 10,500 g/mol, 2.00 g, 0.19 mmol) and purged with dry N_2_. Anhydrous DMSO (15 mL) was added, and the resulting mixture was stirred until complete dissolution of the dextran. Pyridinium *p*-toluenesulfonate (31.2 mg, 0.124 mmol) was added, followed by 2-methoxypropene (6.8 mL, 74 mmol). The flask was placed under an N_2_ environment, then sealed to prevent the evaporation of 2-methoxypropene. After 3 h, the reaction was terminated with TEA (2 mL, 14 mmol), and the modified dextran was precipitated in dd-H_2_O (200 mL). The product was isolated by centrifugation at 5000 rpm for 10 min, and the resulting pellet was washed thoroughly with Milli-Q water (4 × 50 mL, pH 8) by vortexing and sonication followed by centrifugation and removal of the supernatant. Residual water was removed by lyophilization, yielding Ac-DEX (1.99 g) as a fine white powder.

Sp-Ac-DEX was obtained by a three-step chemistry treatment according to the previous literature [30]. (1) Partial Oxidation of Dextran: Dextran (5.0 g, 0.48 mmol, M_W_ = 10 500 g/mol) was dissolved in 20 mL water. After adding sodium periodate (1.1 g, 5.14 mmol), the solution was stirred for 5 h at room temperature. The product was purified by dialysis of the solution against distilled water using a regenerated cellulose membrane with a molecular weight cut off of 3500. The water was changed five times and the sample was lyophilized to obtain a white powder. (2) Acetalation of Partially Oxidized Dextran: Briefly, 3.0 g of partially oxidized dextran (0.28 mmol) was modified with 2-methoxypropene (10.6 mL, 111 mmol), yielding partially oxidized acetalated dextran. (3) Spermine-Modified Ac-DEX: Partially oxidized Ac-DEX (2.0 g, 0.19 mmol) was stirred with spermine (4.0 g, 19.8 mmol) in 10 mL DMSO at 50 °C for 22 h. The reduction was performed for 18 h at room temperature by adding NaBH_4_ (2.0 g, 52.9 mmol) to the DMSO solution. The spermine-modified dextran was precipitated in Milli-Q water (40 mL). The product was isolated by centrifugation at 5000 rpm for 10 min, and the resulting pellet was washed thoroughly with Milli-Q water (5 × 40 mL, pH 8) by resuspension followed by centrifugation and removal of the supernatant. Residual water was removed by lyophilization, yielding spermine-functionalized acetalated dextran spermine-Ac-DEX (1.4 g) as a white powder.

PLGA (M_w_ 76,000–115,000, lactide/glycolide 75:25, ester-terminated, Sigma-Aldrich, Helsinki, Finland) and chitosan (M_w_ 50,000-190,000, Sigma-Aldrich) were used as purchased from the supplier without further treatment.

### 2.3. Fabrication of Polymeric Nanoparticles (NPs)

**Figure 2:**
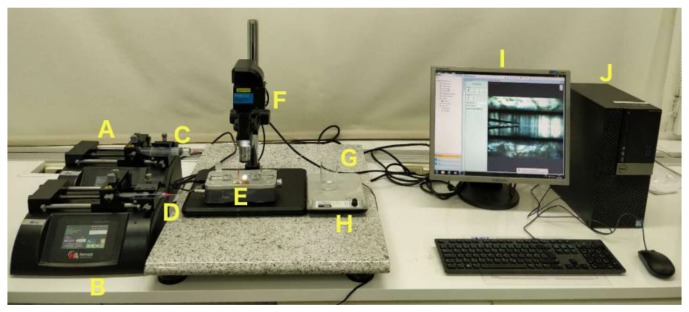
Workstation to fabricate polymer into nanoparticles (NPs). (**A**) Pump A; (**B**) Pump B; (**C**) Syringe A; (**D**) Syringe B; (**E**) Microfluidics chip; (**F**) Microscope; (**G**) Beaker; (**H**) Stirrer; (**I**) Monitor; (**J**) Computer.

**Figure 3:**
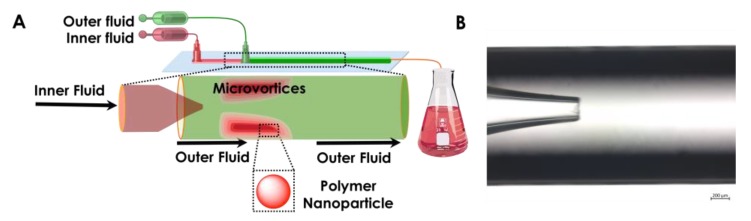
(**A**) A schematic representation of 3D co-flow microfluidics and (**B**) a digital view of the inner and outer capillary.

The nanoprecipitation of polymer into particles was accomplished by following our previous protocol [31]. As shown in Figure 2, two miscible liquids, for example, an aqueous solution of a surfactant and an ethanol solution of the polymer, were filled into syringe A and syringe B. Then, the liquids were injected by pump A and pump B into the microfluidics chip through polyethylene tubes connected to syringes by a needle (20 G) at constant flow rates. The polymer in ethanol served as the inner dispersed phase; meanwhile, a Pluronic^®^ F-127 (Sigma-Aldrich, Helsinki, Finland), 0.1% aqueous solution was selected as the outer continuous fluid. The inner and the outer fluids were independently pumped into the microfluidics device, in which the inner fluid was focused by the outer continuous fluid as shown in Figure 3A. The organic phase exited as a jet from the tapered opening of the inner capillary and quickly mixed in the outer capillary (Figure 3B) with the anti-solvent.

The flow rates of the different liquids were controlled by pumps (PHD 2000, Harvard Apparatus, Holliston, MA, USA). In this procedure, the water-insoluble polymer self-assembled into NPs during its diffusion from the ethanol solution into water under proper conditions. In order to optimize the physicochemical properties of the prepared NPs, including the particle size, polydispersity index (PDI), and zeta (z)-potential, several process variables and formulation parameters were evaluated, such as the capillary fluid rate, the flow ratio between the inner and outer fluids, and the concentration of applied polymer in the inner capillary.

### 2.4. Characterization of Polymeric NPs

Particle sizing was carried out using dynamic light scattering with a Zetasizer Nano ZS (Malvern Instruments Ltd., Worcestershire, UK). For each measurement, the sample suspension without further treatment (~1.0 mL) was put in a disposable polystyrene cuvette (SARSTEDT AG & Co., Nümbrecht, Germany). The nanocarrier surface ζ-potential was measured using a Zetasizer Nano ZS by using disposable folded capillary cells (DTS1070, Malvern Instruments, Malvern, UK). Both the size and ζ-potential were recorded as the average of three measurements. The structure of the fabricated nanoparticle was evaluated by transmission electron microscopy (TEM; JEOL 1400 Plus, JEOL, Tokyo, Japan) at an acceleration voltage of 120 kV. The TEM samples were prepared by depositing 10 µL of the NP suspensions (~1.0 mg/mL) onto carbon-coated copper grids (200 mesh; Ted Pella, Inc., Redding, CA, USA). Samples were blotted away after 5 min incubation then air-dried prior to imaging.

## 3. Results

The Zeta-average diameter and surface potentials (ζ) readout of four types of NPs from DLS was co-related with the polymer concentration, flow rates, and inner tapered capillary opening. In general, the higher the polymer concentration and faster the outer flow rate, the larger the particle size and broader the polydispersity index (PDI) distribution.

**Figure 4:**
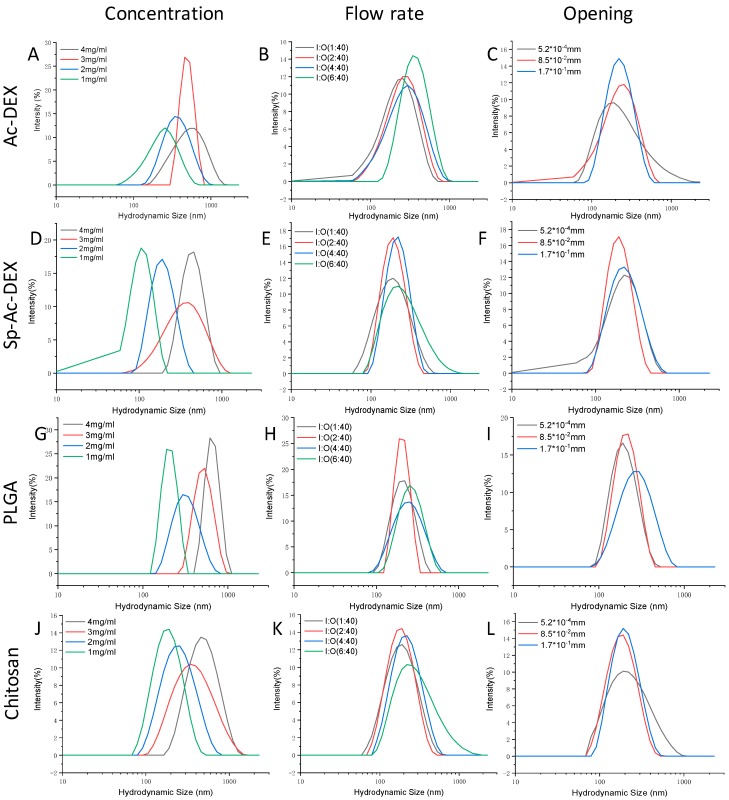
Hydrodynamic diameters of the as-prepared NPs. (**A**) Hydrodynamic sizes at different acetalated dextran (Ac-DEX) concentrations with fixed I/O flow at 2:40 mL/h (8.5 × 10^−^^2^ mm) in the micro-channels; (**B**) Hydrodynamic sizes at a 1 mg/mL Ac-DEX concentration with different fixed I/O flows (8.5 × 10^−2^ mm) in the micro-channels; (**C**) Hydrodynamic sizes at 1 mg/mL Ac-DEX concentration with fixed I/O flow at 1:40 mL/h in different micro-channels; (**D**) Hydrodynamic sizes at different spermine acetalated dextran (Sp-Ac-DEX) concentrations with fixed I/O flow at 2:40 mL/h (8.5 × 10^−2^ mm) in the micro-channels; (**E**) Hydrodynamic sizes at 2 mg/mL Sp-Ac-DEX concentration with different I/O flows (8.5 × 10^−2^ mm) in the micro-channels; (**F**) Hydrodynamic sizes at 2 mg/mL Sp-Ac-DEX concentration with fixed I/O flow at 2:40 mL/h in different micro-channels; (**G**) Hydrodynamic sizes at different poly(lactic-co-glycolic acid) (PLGA) concentrations with fixed I/O flow at 2:40 mL/h (8.5 × 10^−2^ mm) in the micro-channels; (**H**) Hydrodynamic sizes at 1 mg/mL PLGA concentration with different I/O flows (8.5 × 10^−2^ mm) in the micro-channels; (**I**) Hydrodynamic sizes at 1 mg/mL PLGA concentration with fixed I/O flow at 1:40 mL/h in different micro-channels; (**J**) Hydrodynamic sizes at different chitosan concentrations with fixed I/O flow at 2:40 mL/h (8.5 × 10^−2^ mm) in the micro-channels; (**K**) Hydrodynamic sizes at 1 mg/mL chitosan concentration with different I/O flows (8.5 × 10^−2^ mm) in the micro-channels; (**L**) Hydrodynamic sizes at 1 mg/mL chitosan concentration with fixed I/O flow at 2:40 mL/h in different micro-channels.

When we fixed the inner/outer (I/O) fluid flow ratio at 2:40 and the inner opening at 8.5 × 10^−2^ mm (Figure 4A), changing the concentration of Ac-DEX in the inner flow lead to variation in the hydrodynamic size and corresponding PDI. For instance, at the highest Ac-DEX level of 4 mg/mL, an average particle size of 559.1 nm and PDI of 0.308 were recorded. A decrease in the Ac-DEX concentration could minimize the particle size and improve the particle PDI. When the Ac-DEX level in the inner capillary was decreased from 3 to 2 mg/mL, the particle size diminished from 454.5 to 334.0 nm. Further dilution of the polymer solution to 1 mg/mL resulted in a smaller particle size of 227.0 nm and PDI of 0.164, demonstrating that the concentration of Ac-DEX plays a key role in the size of NPs and its distribution. Similarly, the I/O fluid flow is also an important factor. The average size of the Ac-DEX NPs at an I/O fluid flow of 1:40 was 195.7 nm with a PDI of 0.184 at a fixed Ac-DEX concentration of 1 mg/mL, while the average size was 278.3 nm with a PDI of 0.210 when we increased the I/O fluid flow up to 4:40. A further increase of the inner fluid flow increased the particle size up to 336.0 nm. The effect of the opening of the tapered inner capillary on the particle size was also investigated. When the opening was 5.2 × 10^−4^ mm, the fluid flow was 1:40, and the Ac-DEX concentration was 1 mg/mL, the average diameter of the fabricated NPs was 201.2 nm with a PDI of 0.230. Increasing the opening to 8.5 × 10^−2^ mm without changing the other operational conditions gave 195.7 nm NPs with a PDI of 0.194, and doubling the opening size to 1.7 × 10^−1^ mm gave an NP diameter and PDI of 210.6 nm and 0.198, respectively. This implies that the opening of the tapered inner capillary has a minor effect on the particle size, which is consistent with a previous report by Bárbara et al. [32]. The ζ of the as-prepared Ac-DEX NPs was between −5.2 and −8.8 mV (Table 2), showing that the net electrical charge of these NPs is negative. The larger particles are associated with higher absolute ζ potential, indicating that the particles are instable and tend to aggregate.

Sp-Ac-DEX, the amination product of Ac-DEX, was first synthesized for the delivery of siRNA therapeutics [30]. The same protocol was applied to produce Sp-Ac-DEX NPs. Similar to the Ac-DEX NPs, the size and polydispersity of the Sp-Ac-DEX NPs were also dependent on the polymer concentration (Figure 4D), I/O fluid flow ratio (Figure 4E), and the opening (Figure 4F) of the tapered inner capillary. However, the size of the Sp-Ac-DEX NPs was generally smaller and the PDI was statistically narrower (Table 2) compared to those of the Ac-DEX NPs.

PLGA, an US Food and Drug Administration approved polymer, is biodegradable, biocompatible, and tunable in terms of its mechanical properties; therefore, it remains the most attractive polymeric candidate in use for biomedical applications [33,34]. The methods used to produce PLGA-based NPs, represented by emulsification solvent evaporation/diffusion, coacervation, emulsification reverse salting-out, dialysis, spray drying, and nanoprecipitation methods, have been reviewed [35]. We used the in-house-built chip to produce the NPs by the nanoprecipitation method. As the DLS graph in Figure 4G–I shows, the obtained PLGA NPs are like those from Ac-DEX and PLGA under the same conditions, but a more uniform distribution is evidenced when compared to Ac-DEX and SP-Ac-DEX. For instance, when 4 mg/mL PLGA ethanol solution was injected with an I/O flow rate of 2:40 (8.5 × 10^−2^ mm) into the micro-channels, a 611.9 nm particle diameter was given, while Ac-DEX and Sp-Ac-DEX only gave particles of 559.1 nm and 409.5 nm in diameter (Table 2) individually under the same operational conditions. However, the ζ values of the PLGA NPs approach zero, implying that the NPs produced in this way are unstable.

Chitosan is a linear polymer naturally occurring only in Mucoraceae and is chemically composed of glucosamine and *N*-acetylglucosamine monomers linked through β-(1−4)glycosidic linkages [36]. Primary C-6 OH and secondary C-2 NH_2_ groups offer chitosan easy access for modification; hence, a variety of biomedical applications have been found, exemplified by the investigation of its structure–activity relationship with antibiotics [37]. It has been also used as a coating material to encapsulate astaxanthin into nanosized composite for enhanced water solubility, stability, and bioavailability [38]. To demonstrate the generality of our nanoprecipitation approach, chitosan nanoparticles were also produced. As showed in Figure 4J–L, chitosan particles are smaller than those of Ac-DEX and PLGA and of equal size to Sp-Ac-DEX-based ones in terms of diameter. The ζ values of chitosan NPs are between 14.0 and 15.3 (Table 2), implying improved stability when compared to Ac-DEX and Sp-Ac-DEX.

**Figure 5:**
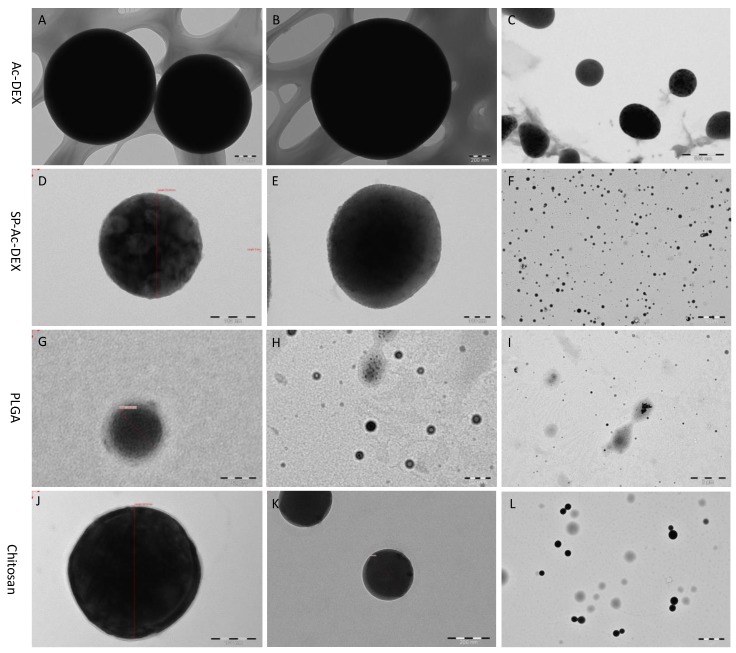
TEM images of as-prepared NPs. (**A**–**C**): Ac-DEX; (**D**–**F**): Sp-Ac-DEX; (**G**–**I**): PLGA; (**J**–**L**): chitosan. Scale bars: (**D**,**E**,**J**): 100 nm; (**A**,**B**,**K**): 200 nm; (**C**,**G**,**H**): 500 nm; (**L**) 1 μm; (**I**) 2 μm; (**F**) 5 μm.

TEM showed that all the experimental polymers—Ac-DEX, SP-Ac-DEX, PLGA, and chitosan—form spherical-like NPs. However, they present different sphericalities. As shown in Figure 5A,E,I, the dark sphere and blurred underlay demonstrate that not all Ac-DEX forms NPs. In the case of the other materials, it is rare to see unmolded polymer NPs, although the TEM images of PLGA NPs are low in contrast (Figure 5C,G,K).

## 4. Discussion

The microfluidics chip presented in this experiment is easy to assemble. One experienced researcher can produce a device within 10 min. Most importantly, the main materials used for the chip assembly are glass and minor metal; therefore, they are fully resistant to organic solvents. The epoxy resins for solidification have little chance of contact with the organic solvent. Consequently, the channel of the chip has no problems with swelling and deformation and therefore offers more reliable flow rate and pressure. What we showed here is the prototype of the microfluidics chip we developed several years ago [16]. In fact, this droplet microfluidics device has been dramatically diversified and upgraded, such as for single-cell analysis tools, small-scale cell cultures, in-droplet chemical synthesis, high-throughput drug screening, and nanodevice fabrication [39]. In particular, when two immiscible phases, such as water and ethyl acetate or water and ethyl carbonate, go in a flow focus manner, W/O or O/W emulsion [40]—or even a W/O/W double emulsion [41,42]—with the desired profile can form depend on the properties of the surfactants. No matter how complex the emulsion structure is, the basic elements are the same. Besides this, more than two channels can also be integrated into one device to form multiplexed microfluidics to match advanced requirements; for instance, superfast sequential microfluidics nanoprecipitation for paclitaxel nanocrystal core formation and drug encapsulation with a polymer can be performed using one microfluidics chip by combining three phases together [43].

Regardless of the complexity of the obtained particles/droplets from microfluidics, the Reynolds number (*Re*) remains a key parameter in this process; it defines the flow pattern in microfluidics channels and can be defined by the following Equation:

Re=ρULoμ= ρQμE

where ρ is the density of the fluid, U is the velocity of flow, μ is the viscosity of the fluid, and E is the channel diameter of the capillary. For a given microfluidics chip, high viscosity of the liquids or density of the fluids, or a fast flow rate, will lead to higher *Re*. Increased *Re* dictates changes in the mixing patterns of the fluids inside a microchannel from laminar flow to microvortices, even to jetting, also implying variation in the size and polydispersity of the NPs. In the current system, the enhanced mixing of two miscible phases is desired with the aim to obtain smaller sized NPs for future drug encapsulation application. Therefore, laminar flows related mixing need to avoid in this experiment. Of course, the robustness of this platform offers the possibility of larger size, such as microparticle production as well, if needed.

The enlargement of the NPs with increasing polymeric concentration is understandable since more starting material input will enhance the probability of clustering between as-prepared NPs [44]. The minor size increase of the NPs with increasing I/O fluid flow ratio can be accounted for as follows: (1) In a sense, the increased inner capillary flow is identical to increased polymeric concentration and therefore leads to bigger NP size. (2) Increasing the I/O will decrease the focusing degree [45] when the outer flow is fixed, while increasing the *Re* favors vortex ring formation [46]. The vortex formation helps the split of bigger primary vortex rings into smaller secondary or tertiary vortex rings [47], which could correlate with smaller NP output. However, the enhanced polymeric input due to the increased inner capillary flow and dispersed focusing degree may offset and even outnumber the effect of increased *Re* on decreased NP size in experimental conditions; therefore, an overall increased NP size result.

The as-prepared NPs inherited their physiochemical properties from the parent polymeric material itself. The positive electrical surface charge of the Sp-Ac-DEX NPs originates from the spermine group, which is a linear oligamine with a p*K*a of 11.1 (DrugBank); therefore, Sp-Ac-DEX is prone to being protonated under 1× PBS buffer. The Ac-DEX NPs have negative net electrical charge; chemically, Ac-DEX NPs are neutral, but the negative ζ value may be because they specifically absorb negatively charged ions. The absolute ζ value of Sp-Ac-DEX NPs is higher [44] than that of Ac-DEX NPs (Table 2), which implies that the protonation effect outweighs the absorption of negatively charged ions on the polymer. The attachment of spermine to Ac-DEX enhances its hydrophilicity, which in return favors the self-assembly of the polymer into NPs and is more sensitive to polymer concentration variation. In addition, the affinity of Sp-Ac-DEX to cells is also improved when compared to Ac-DEX since positively charged NPs can penetrate deep into cell membranes [48]. The positive ζ value of chitosan NPs comes from the protonation of C-2 NH_2_ but is lower than that of Sp-Ac-DEX NPs, which may relate with the lower p*K*a of the chitosan C-2 NH_2_ group compared with that of spermine.

It is intuitively understandable that a high polymer concentration will lead to larger NPs due to more mass transference occurring in nanoprecipitation. It is worth pointing out that a narrow NP distribution and the desired particle size (often smaller ones) are not always simultaneously achievable. For instance, injection of 3 mg/mL Ac-DEX into microfluidics devices gives NPs with a PDI of 0.112—the lowest result among all the experimental polymer concentrations. This may suggest that the concentration of the polymer is not the only factor determining polymer precipitation. Under a fixed concentration of polymer, increasing the flow rate of the inner capillary is intuitively equal to increasing the input of polymer and also changes the mixture pattern of inner and outer flow in the outer capillary, leading to the variation of the NP morphology. The Sp-Ac-DEX- and PLGA-made NPs are not as sensitive as Ac-DEX and chitosan NPs to the variation of the inner capillary flow rate; a possible explanation for this is that greater polymer input (higher polymer concentration) relates with larger particle size while a fast phase mixture offers smaller particles with a narrow size distribution. Under fixed pump pressure, a smaller inner capillary opening is related with higher kinetic motion of the inner phase solvent according to Bernoulli’s principle, leading to fast solvent transfer and polymer precipitation. The insensitivity of polymeric NPs to the opening in our experiment is hard to rationalize since the formation of NPs depends on multiple factors, and it is possible they offset each other to obtain the desired polymeric NPs.

## 5. Conclusions

Herein, we detailed the protocol used to fabricate a novel flow-focusing microfluidics chip mainly based on glass material. The easy-to-handle profile of this chip allows its potential scalability into industrialization. The purpose of this glass lab-on-a-chip droplet generator is to fabricate NPs. The robustness of this platform for solvent replacement nanoprecipitation was demonstrated by the preparation of Ac-DEX, Sp-Ac-DEX, PLGA, and chitosan NPs. Three parameters expected to have an effect on the morphology of the NPs were investigated. The order of their contribution is as follows: polymer concentration > I/O flow rate > the opening of the inner capillary.

## Figures and Tables

**Table 1:** The materials and accessories used to assemble the microfluidics chip.

Material	Specification	Amount	Producer
Glass slide	75 × 25 mm	1	BrandTech (Essex, CT, USA)
Outer capillary	OD^a^ = 1.5 mm; ID^b^ = 1.12 mm	1	World Precision Instruments, Inc (Sarasota, FL, USA)
Inner capillary	OD^a^ = 1.0 mm; ID^b^ = 0.58 mm	1	World Precision Instruments, Inc (Sarasota, FL, USA)
Syringe tip	Blunt end needle	3	Warner Instruments (Hamden, CT, USA)
Glue	5 min Epoxy	---	Devcon (Danvers, MA, USA)
Puller	Model PN-31	1	Narishige (Tokyo, Japan)
Sandpaper	Grit = 1200 Only a small piece	1	Indasa–Rhynowet (Aveiro, Portugal)
Diamond tip glass cutter	---	1	Harden (Xi’an, China)

OD^a^ = outer diameter; ID^b^ = inner diameter.

**Table 2:** The PDI values and surface potentials of as-prepared NPs under different conditions ^a^.

Conditions	Ac-DEX		Sp-Ac-DEX		PLGA		Chitosan	
4 mg/mL	0.308	−8.8	0.214	16.2	0.160	1.2	0.179	14.3
3 mg/mL	0.112	−7.9	0.253	15.5	0.186	0.9	0.211	12.5
2 mg/mL	0.178	−7.1	0.100	15.1	0.198	−1.3	0.249	11.9
1 mg/mL	0.164	−5.7	0.241	13.9	0.088	0.5	0.175	12.1
1:40	0.184	−5.2	0.197	14.2	0.059	−0.8	0.163	11.2
2:40	0.164	−5.7	0.100	15.1	0.088	0.5	0.175	12.1
4:40	0.210	−6.3	0.097	16.1	0.123	0.3	0.176	11.8
6:40	0.173	−7.4	0.183	16.6	0.239	0.7	0.203	12.8
5.2 × 10^−4^ mm	0.194	−6.1	0.177	15.6	0.135	0.9	0.215	11.6
8.5 × 10^−2^ mm	0.184	−5.2	0.100	15.1	0.059	−0.8	0.175	12.1
1.7 × 10^−1^ mm	0.198	−6.5	0.175	16.2	0.134	−1.2	0.125	12.0

^a^ The data are arranged such that the first and second subcolumns of each main column represent the PDI and surface potential with a unit of mV, respectively**.**
