# Correction: Ma, X. et al. Conducting Polymeric Nanocomposites with a Three-Dimensional Co-flow Microfluidics Platform. *Micromachines*, 2019, *10*, 383

**DOI:** 10.3390/mi10090604

**Published:** 2019-09-12

**Authors:** Xiaodong Ma, Yuezhou Zhang, Korbinian Weisensee

**Affiliations:** 1 Xi’an Institute of Flexible Electronics & Xi’an Institute of Biomedical Materials and Engineering, Northwestern Polytechnical University (NPU), Xi’an 710072, China; 13851280968@163.com; 2 Department of Pharmaceutical Science Laboratory, Åbo Akademi University, 20520 Turku, Finland; zyuezhou@126.com

The authors would like to indicate the following financial support they received to the Funding Section of their published paper [1]: “This research was supported by the Fundamental Research Funds for the Central Universities of China, grant number 31020190QD030.”

The changes do not affect the scientific results. We apologize for any inconvenience caused to the readers. The manuscript will be updated, and the original will remain online on the article webpage, with a reference to this correction.
